# Investigating Volcanic Plumes from Mt. Etna Eruptions of December 2015 by Means of AVHRR and SEVIRI Data

**DOI:** 10.3390/s19051174

**Published:** 2019-03-07

**Authors:** Francesco Marchese, Alfredo Falconieri, Carolina Filizzola, Nicola Pergola, Valerio Tramutoli

**Affiliations:** 1 Institute of Methodologies for Environmental Analysis (IMAA), Italian Research Council (CNR), 85050 Tito Scalo (PZ), Italy; alfredo.falconieri@imaa.cnr.it (A.F.); carolina.filizzola@imaa.cnr.it (C.F.); nicola.pergola@imaa.cnr.it (N.P.); 2 School of Engineering, University of Basilicata, 85100 Potenza, Italy; valerio.tramutoli@unibas.it

**Keywords:** Mt. Etna, ash clouds, AVHRR, SEVIRI

## Abstract

In early December 2015, a rapid sequence of strong paroxysmal events took place at the Mt. Etna crater area (Sicily, Italy). Intense paroxysms from the Voragine crater (VOR) generated an eruptive column extending up to an altitude of about 15 km above sea level. In the following days, other minor ash emissions occurred from summit craters. In this study, we present results achieved by monitoring Mt. Etna plumes by means of RST_ASH_ (Robust Satellite Techniques-Ash) algorithm, running operationally at the Institute of Methodologies for Environmental Analysis (IMAA) on Advanced Very High Resolution Radiometer (AVHRR) data. Results showed that RST_ASH_ detected an ash plume dispersing from Mt. Etna towards Ionian Sea starting from 3 December at 08:40 UTC, whereas it did not identify ash pixels on satellite data of same day at 04:20 UTC and 04:40 UTC (acquired soon after the end of first paroxysm from VOR), due to a mixed cloud containing SO_2_ and ice. During 8–10 December, the continuity of RST_ASH_ detections allowed us to estimate the mass eruption rate (an average value of about 1.5 × 10^3^ kg/s was retrieved here), quantitatively characterizing the eruptive activity from North East Crater (NEC). The work, exploiting information provided also by Spinning Enhanced Visible and Infrared Imager (SEVIRI) data, confirms the important contribution offered by RST_ASH_ in identifying and tracking ash plumes emitted from Mt. Etna, despite some operational limitations (e.g., cloud coverage). Moreover, it shows that an experimental RST product, tailored to SEVIRI data, for the first time used and preliminarily assessed here, may complement RST_ASH_ detections providing information about areas mostly affected by volcanic SO_2_.

## 1. Introduction

**Figure 1:**
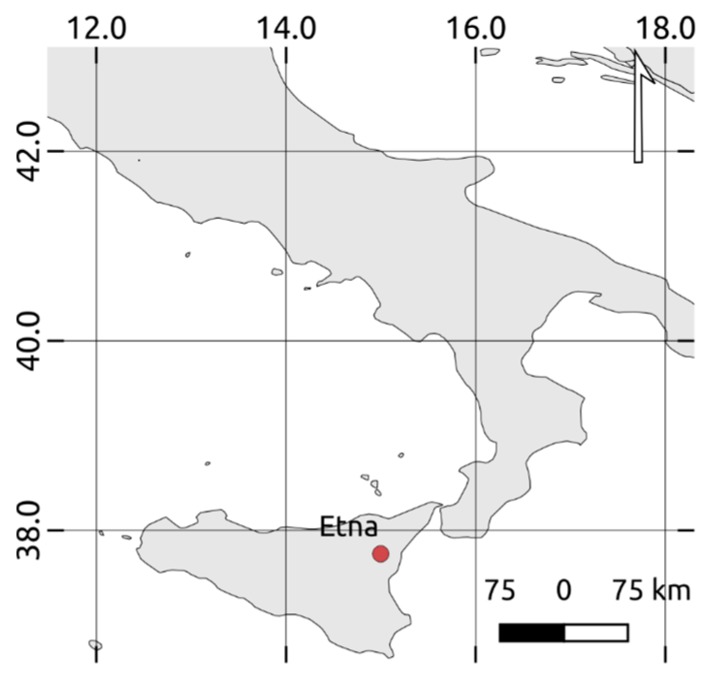
Geographic location of Mt. Etna (Sicily, Italy).

Mt. Etna (Sicily-Southern Italy; see Figure 1) is the most active volcano in Europe. Frequent eruptions (e.g., gas/ash emissions, Strombolian activities, lava fountains, lava flows) characterize this stratovolcano (e.g., [1]). Eruptions may occur both at summit craters, namely Voragine (VOR), Bocca Nuova (BN), North East Crater (NEC) and New Southeast Crater (NSEC), and on its flanks where lava effusions pose the major risk for surrounding populated areas (e.g., [2]).

**Figure 2:**
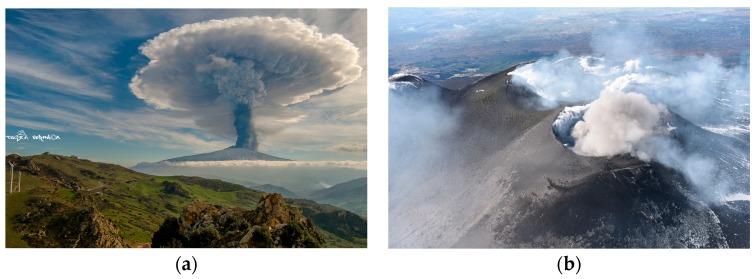
(**a**) Mt. Etna eruptive column of 4 December 2015, photograph courtesy of Veronica Testa; (**b**) Explosion from North East Crater (NEC) of 14 December 2015, photograph courtesy of Marco Neri.

In recent years, a long series of powerful paroxysmal events took place at the crater area, revealing a general increment in the explosive activity [2]. Among those eruptions, the four paroxysms from VOR of 3–5 December 2015 were particularly intense (e.g., [3,4,5]). The first one started on 3 December at around 02:30 UTC, generating an eruptive column extending up to 15 km above sea level (a.s.l.). This eruptive event ended at 03:30 UTC, causing the ashfall also in Reggio Calabria (Southern Italy), which is located about 70 km from the volcanic area [3,4,5]. In the following hours, sporadic ash emissions took place at the BN and NSEC [4]. The day after (see Figure 2a), the second and third paroxysmal events occurred (during 9:10–10:10 UTC and 20:30–21:10 UTC, respectively), generating an eruptive column reaching about 13.4 km a.s.l. [5]. In addition, a new pit crater opening up on the East flank of the NSEC also emitted ash [4,5]. On 5 December, the volcano entered into the fourth paroxysmal phase, which lasted about one hour (i.e., from 14:50 UTC to 15:40 UTC). After those major eruptive events, NSEC activated emitting, during 6–8 December, a plume of fine ash (Strombolian explosions and a lava effusion also occurred from this crater). Almost concurrently, weak to moderate ash emissions took place at the NEC, first gradually increasing (i.e., after the end of the NSEC eruption) and then continuing at a decreased rate until 11 December [4]. In the late afternoon of 13 December, NSEC emitted once again ash. In the following two days, intermittent ash emissions took place at the NEC (e.g., see Figure 2b). Finally, after two-short lived events recorded at the VOR, minor ash emissions from NSEC occurred on 28 December [4].

Volcanic ash from Mt. Etna eruptive activity of 3 December caused a temporary air traffic disruption at both Catania (Sicily, Italy) and Reggio Calabria (Southern Italy) airports [6,7]. Besides, a significant amount of volcanic sulfur dioxide, travelling eastwards, was also emitted [8]. Recent studies investigated the intense paroxysms from VOR also by means of satellite observations, retrieving information about features of emitted plume (e.g., [4,8,9,10,11]).

In this work, we present results of ash detections achieved, during the different phases of December 2015 eruptions, by using the RST_ASH_ algorithm [12] running operationally on NOAA (National Oceanic and Atmospheric Administration) and MetOp (Meteorological Operational Satellites)-AVHRR (Advanced Very High Resolution Radiometer) data acquired and processed at IMAA (Institute of Methodologies for Environmental Analysis) [13]. 

To characterize the eruptive activity from NEC, estimates of plume height and mass eruption rate are also shown and discussed. In addition, an experimental RST-based product tailored to SEVIRI (Spinning Enhanced Visible and Infrared Imager) data is, for the first time assessed here, verifying its potential in mapping SO_2_-affected areas, as a complement to existing satellite-based products used for detecting and monitoring volcanic plumes. 

## 2. Data

The AVHRR is a multi-spectral sensor designed for meteorological studies. This instrument, having a radiometric resolution of 10-bit, provides data in five spectral channels ranging from VIS (Visible) to TIR (Thermal Infrared) bands; channel 3 of AVHRR/3 was split into channel 3A (1.58–1.64 µm), working during daytime, and channel 3B (3.55–3.93 µm) operating at nighttime. The spatial resolution is equal to 1.1 km at the nadir view, and the temporal coverage is of about 6 h, due to two platforms operating in pair. 

Channel 4 (10.3–11.3 µm) and channel 5 (11.5–12.5 µm) have largely been used for ash detection purposes, by exploiting the reverse absorption effects of silicate particles at 11 and 12 µm wavelengths in comparison with water droplets and ice (e.g., [14,15,16,17,18]). Besides, a number of studies showed that channel 3 (i.e., channel 3B of AVHRR/3) located in the MIR (Medium Infrared) region, if used jointly with channel 4 may improve ash identification under different illumination conditions (e.g., [12,16]). 

SEVIRI, onboard MSG (Meteosat Second Generation) geostationary satellites, is a 50 cm-diameter aperture, line-by-line scanning radiometer, providing data every 15 min in 12 different spectral bands (including the High-Resolution Visible channel-HRV) [19]. The spatial resolution of visible and infrared channels is about 3 km at the nadir (1 km for the HRV channel). Among those channels, band 7 centered in the TIR band at 8.7 µm wavelength (8.3–9.1 µm) was used for volcanic SO_2_ retrieval in the lower troposphere (e.g., [20,21]). In addition, some authors demonstrated that this spectral channel could further increase efficiency of ash detection schemes based on analysis of BTD (Brightness Temperature Difference) signal (e.g., [22]). 

## 3. Methods

### 3.1. RST_ASH_ Algorithm

RST_ASH_ is a specific configuration of the RST (Robust Satellite Techniques) multi-temporal approach [23]. The latter identifies anomalous signal variations, in the space-time domain, by analyzing multi-annual time series of clouds-free satellite records, selected according to some homogeneity criteria (e.g., same spectral channel/s; same hour of day and calendar month). 

RST_ASH_ identifies ash pixels by means of two indices used in combination:

(1) $${⊗}_{BT_{11}-BT_{12}}\left(x,y,t\right)=\frac{(BT_{11}\left(x,y,t\right)-BT_{12}\left(x,y,t\right))-\mu_{BT_{11}-BT_{12}}\left(x,y\right)}{\sigma_{BT_{11}-BT_{12}}\left(x,y\right)},$$

(2) $${⊗}_{BT_{3.7}-BT_{11}}\left(x,y,t\right)=\frac{(BT_{3.7}\left(x,y,t\right)-BT_{11}\left(x,y,t\right))-\mu_{BT_{3.7}-BT_{11}}\left(x,y\right)}{\sigma_{BT_{3.7}-BT_{11}}\left(x,y\right)}.$$

In Equations (1) and (2), $BT_{3.7}\left(x,y,t\right),BT_{11}\left(x,y,t\right),BT_{12}\left(x,y,t\right)$ are the brightness temperatures measured in MIR and TIR bands (subscript number indicates the central wavelength of the considered spectral channel), at the time t and location $\left(x,y\right),\;\mu_{BT_{3.7}-BT_{11}}\left(x,y\right),\;\mu_{BT_{3.7}-BT_{11}}\left(x,y\right)$ are the temporal mean, while $\sigma_{BT_{11}-BT_{12}}\left(x,y\right)$ and $\sigma_{BT_{3.7}-BT_{11}}\left(x,y\right)$ stand for temporal standard deviation of the brightness temperature differences. 

RST_ASH_ indices behave differently in the presence of ash. In particular, negative values of the ${⊗}_{BT_{11}-BT_{12}}\left(x,y,t\right)$ index generally characterize ash clouds, whereas slightly positive values of the ${⊗}_{BT_{3.7}-BT_{11}}\left(x,y,t\right)$ index are usually recorded because of those features, regardless of illumination conditions [13]. 

RST_ASH_ has widely been used to detect ash plumes exploiting both polar (e.g., MODIS-Moderate Resolution Imaging Spectroradiometer) and geostationary (e.g., AHI-Advanced Himawari Imager) satellite data (e.g., [13,24,25,26,27]). The latter guarantee a more efficient monitoring of volcanic phenomena, thanks to the higher temporal sampling (e.g., 10 min in the case of AHI). A recent study has shown that an additional RST_ASH_ index (i.e., ${⊗}_{VIS}\left(x,y,t\right)$), analyzing the visible radiance measured in the SEVIRI channel 1, centered at 0.6 µm wavelength, may be used for better discriminating ash from meteorological clouds in daytime scenes [27]. The operational RST_ASH_ product uses the ${⊗}_{VIS}\left(x,y,t\right)$ index jointly with that of Equation (1) when daytime AVHRR/3 data are processed (the index in Equation (2) cannot be computed in daylight conditions when only channel 3A is available). 

### 3.2. Plume Height and Mass Eruption Rate Estimations

Plume height is a key parameter for numerical models aiming at forecasting ash dispersion in the atmosphere. This parameter, which is also important for performing the aerosol retrieval (e.g., [28]), may be estimated by satellite data using different methodologies, each one of them presenting advantages and drawbacks (e.g., [28,29,30,31,32]). 

In this study, we used the largely accepted cloud-top temperature method to retrieve the plume height starting from RST_ASH_ detections. The method assumes that ash clouds behave as black bodies in thermal equilibrium with the atmosphere. Hence, plume height may be estimated by comparing the minimum brightness temperature of detected ash pixels, i.e., generally that measured at 11 µm, with the relative atmospheric temperature profile (e.g., [33,34]). This procedure generally shows, as the main limitation, a lower accuracy in retrieving the aforementioned parameter in the presence of semi-transparent ash clouds, as well as when airborne ash approaches the tropopause. Moreover, it requires accurate atmospheric temperature profiles for providing optimal results (e.g., [35,36]). 

Starting from estimates of plume height H (km above the vent), we derived the volumetric flow rate V (m^3^ dense-rock equivalent per second) by inverting the empirical formulation reported in [37]:

(3) $$H=2.0\;V^{0.241}.$$

Equation (3), which was derived by using a dataset of historical eruptions, such as other similar empirical formulations (e.g., [38]), provides rough estimates of the volumetric flow rate especially in the presence of ash plumes from small eruptions, which are more significantly affected by windy conditions (e.g., [39,40]). The volumetric flow rate, derived using Equation (3), was then converted into MER (mass eruption rate, kg/s) by multiplying its value by the assumed density of ash (2500 kg/m^3^; e.g., [41]).

## 4. Results

### 4.1. Ash Plume Detection of 3–5 December

**Figure 3:**
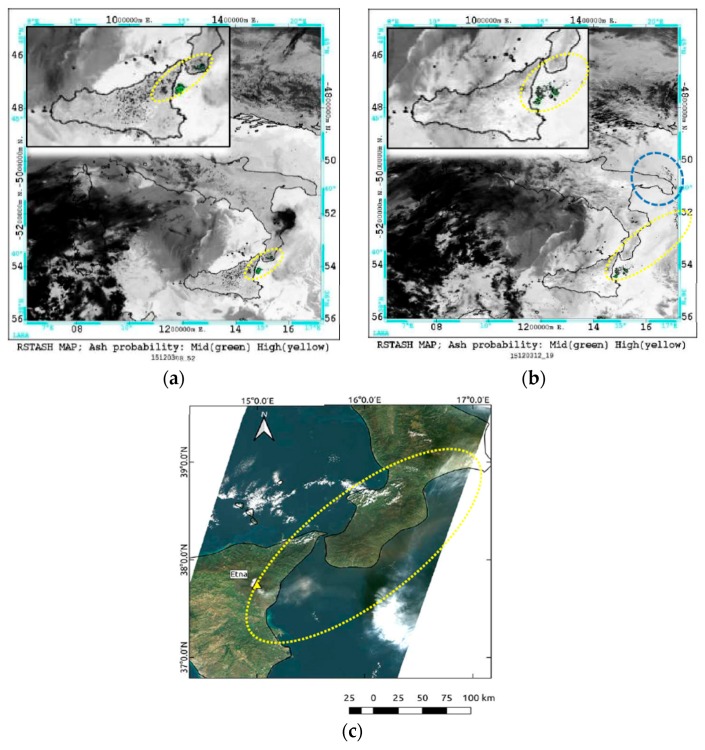
(**a**) Robust Satellite Techniques-Ash (RST_ASH_) map from Advanced Very High Resolution Radiometer (AVHRR) data of 3 December at 08:40 UTC (on the top-left side of the panel, the zoom of detected ash pixels); (**b**) RST_ASH_ map from AVHRR data of 3 December at 12:44 UTC; (**c**) Landsat-8 true color image (Red = Band 4: 0.630–0.680 µm; Green = Band 3: 0.525–0.600 µm, Blue = Band 2: 0.450–0.515 µm) of 3 December at 09:35 UTC.

In this section, we show results of ash detection performed by RST_ASH_ in early December 2015. Figure 3 displays the RST_ASH_ maps (the background shows the brightness temperatures measured in the 11 µm channel), which were automatically generated at IMAA a few minutes after the sensing time, from two consecutive AVHRR overpasses of 3 December. Ash pixels were depicted in two different colors based on confidence levels of detection (i.e., green: low-medium confidence level; yellow: high confidence level). 

In more detail, Figure 3a shows the RST_ASH_ product of 08:40 UTC revealing the presence of an ash plume dispersing from the Mt. Etna crater area towards the Ionian Sea (in the E-NE direction), covering Sicily and part of Calabria region (Southern Italy) (see green pixels within the dotted yellow ellipse). The RST_ASH_ map of Figure 3b, referring to data of 12:44 UTC, showed that the ash plume probably extended up to the coasts of Puglia region (Southern Italy), as indicated by ash pixels flagged within the dotted blue circle. Those maps provided information about the monitored ash phenomenon in good agreement with some independent satellite observations. An example is shown in Figure 3c, displaying the Landsat-8/OLI (Operational Land Imager) true color imagery, at 30 m spatial resolution acquired on 3 December at 09:35 UTC. The figure confirmed that the presence of a thin ash plume (in brown within the area marked by the yellow dotted ellipse) dispersing from the Mt. Etna crater area (see yellow triangle) in the E-NE direction, affecting the coasts of the Calabria region where it appeared slightly more extended than that indicated by RST_ASH_. The latter generated a few artefacts mainly close to the area marked by the yellow ellipse, where the RGB (Red, Green, Blue) product of Figure 3c did not provide any evidence of ash. 

**Figure 4:**
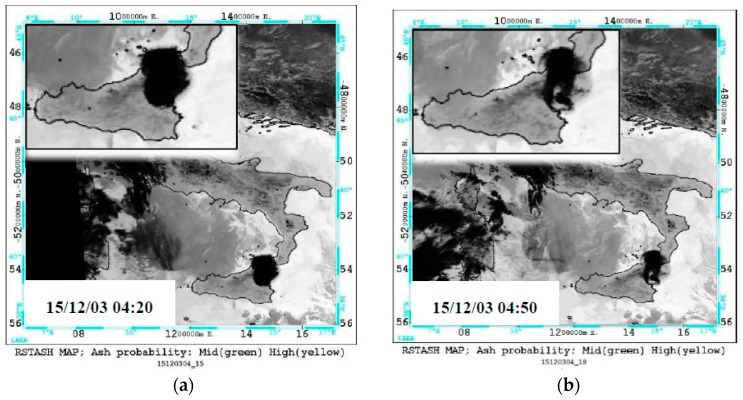
RST_ASH_ maps from AVHRR data of December 3: (**a**) 04:20 UTC; (**b**) 04:50 UTC.

**Figure 5:**
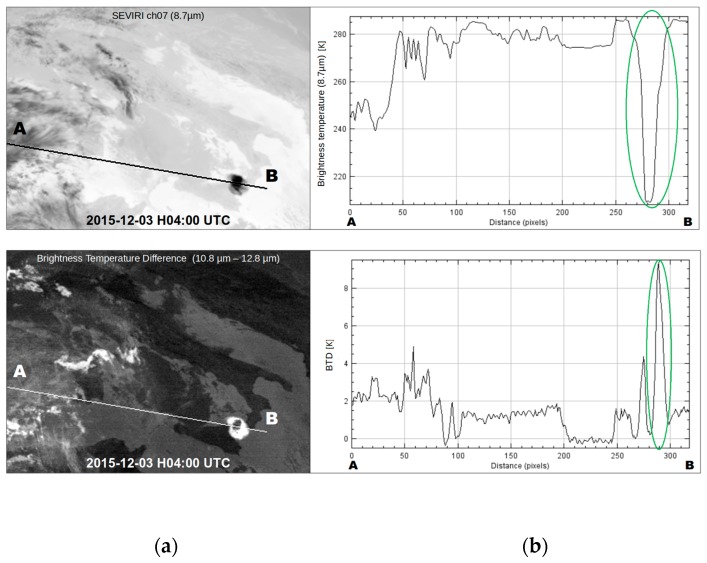
Spinning Enhanced Visible and Infrared Imager (SEVIRI) data of 3 December 2015 at 04:00 UTC: (**a**) Top: Brightness Temperature Difference (BTD) image; Bottom: brightness temperature in the channel 7 (at 8.7 µm); (**b**) signal changes recorded along the A-B transect region. Green ellipse indicate the region affected by the eruption column.

It is worth mentioning, however, that RST_ASH_ maps of 04:20 UTC and 04:50 UTC which were generated from two consecutive AVHRR overpasses acquired soon after the end of first paroxysm from VOR, did not provide any evidence of ash. Figure 4 shows, in fact, that although an eruption cloud possibly affected the monitored geographic area, RST_ASH_ did not flag any ash pixel (see region magnified on top left side of the panels). To investigate factors preventing the identification of airborne ash, we analyzed a number of SEVIRI scenes close in time to available AVHRR observations. Figure 5a displays the BTD (i.e., BT10.8-BT12) and the channel 7 (i.e., centered at 8.7 µm) SEVIRI imagery of 3 December at 04:00 UTC (left panels). The upper panel in Figure 5b shows changes of BTD signal (i.e., 11 µm–12 µm) along the A-B transect region, while the bottom panel displays the BT8.7 variations. As can be seen from the plots, the BTD was mostly positive along the whole analyzed transect region. In particular, it further increased in value (up to about 9 K) over the Mt. Etna area, where a drastic reduction of BT8.7 (from 290 K to about 220 K) was also recorded (see region marked by the green ellipse).

**Figure 6:**
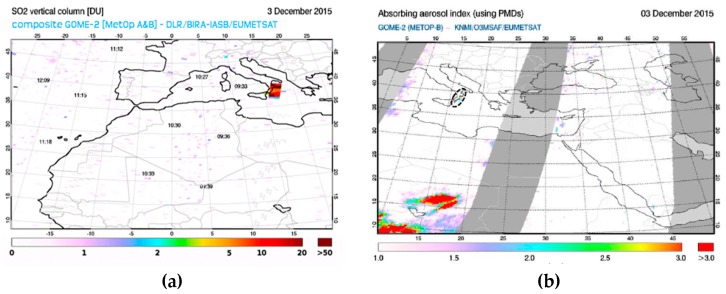
Global Ozone Monitoring Experiment-2 (GOME-2) products of 3 December 2015 made available by Support to Aviation Control Service (SACS) (http://sacs.aeronomie.be/). (**a**) SO_2_ vertical column; (**b**) absorbing aerosol index map.

**Figure 7:**
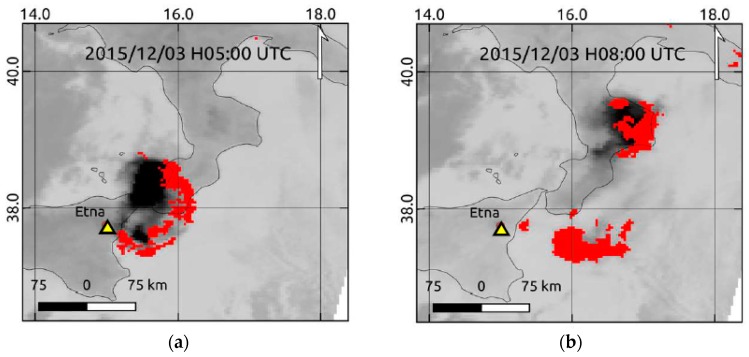

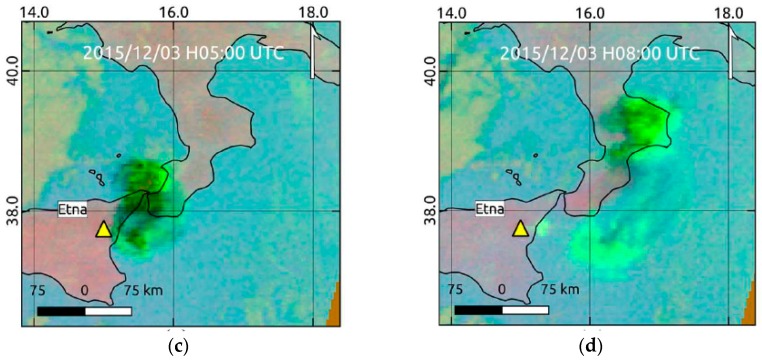
On the top, RST maps from SEVIRI data of 3 December 2015 with ${⊗}_{BT8.7-BT11}\left(x,y,t\right)$ < −2 $AND{⊗}_{BT3.9-BT11}\left(x,y,t\right)$ > 0. In red, areas possibly affected by SO_2_ (**a**) 05:00 UTC, (**b**) 08:00 UTC. On the bottom, ash RGB product generated according to the EUMETSAT scheme (i.e., Red: BT12.0-BT10.8: −4.0 to 2.0 K; BT10.8-BT8.7: −4.0 to 5.0 K; BT10.8: 243 to 343 K); in green the areas affected by SO_2_, in blue thin ice (**c**) 05:00 UTC, (**d**) 08:00 UTC.

Hence, while the BTD reached values generally associated with water/ice clouds, the BT8.7 strongly decreased over the plume area, probably because of volcanic sulfur dioxide (SO_2_ strongly absorbs at 8.7 µm wavelength; e.g., [20]). This hypothesis fits with results of previous studies (e.g., [42,43]) as well as with information provided by the SO_2_ vertical column product from GOME-2 (Global Ozone Monitoring Experiment-2) data of 3 December, made freely available online by SACS (Support to Aviation Control Service) [44]. Figure 6a shows, in fact, that a few hours after the end of the first paroxysm from VOR, a SO_2_ plume affected both the Ionian Sea and Calabria region. In that period, ash coverage was less evident, as indicated by the GOME-2 absorbing aerosol index map of Figure 6b (see region marked by the dotted black ellipse). Moreover, by analyzing the BT8.7-BT10.8 signal in the space-time domain, according to the general RST scheme, we found that pixels with negative values of ${⊗}_{BT_{8.7}-BT_{11}}\left(x,y,t\right)$ index, defined using the same approach as in Equations (1) and (2), affected the same SO_2_ areas flagged by the ash RGB product from EUMETSAT (European Organization for the Exploitation of Meteorological) [45]. This product provides qualitative information about ash (reddish colors) and SO_2_ (in shades of bright green) plumes dispersion, combing three SEVIRI infrared bands; i.e., BT12.0-BT10.8 (Red), BT10.8-BT8.7 (Green) and BT10.8 (Blue) [46]. Specifically, red pixels in Figure 7 top panels, i.e., those having values of ${⊗}_{{\mathrm{BT}}_{8.7}-\mathrm{BT}11}\left(x,y,t\right)$ < −2 *AND* ${⊗}_{{\mathrm{BT}}_{3.9}-\mathrm{BT}11}\left(x,y,t\right)$ > 0 (index in Equation (2) was used for better filtering meteorological clouds), were in spatial agreement with the green ones in the bottom panels indicating the presence of volcanic SO_2_ (dark blue pixels indicated thin ice [46]). In addition, SO_2_ areas in Figure 7d were consistent with those of Figure 6a. Therefore, the experimental RST product provided information about spatial dispersion of volcanic SO_2_ in good agreement both with the ash RGB product from SEVIRI data and with independent GOME-2 observations from SACS. 

During 4–7 December, although other eruptions took place at the Mt. Etna crater area, RST_ASH_ did not detect ash mostly because of a thick cloud coverage. Nevertheless, during 8–10 December it successfully identified and tracked the ash plume, as shown and discussed in the next section.

### 4.2. Ash Plume Detection of 8–10 December

**Figure 8:**
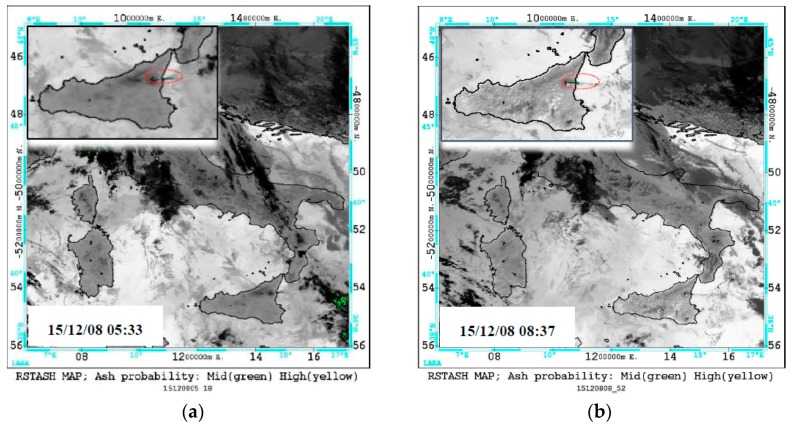

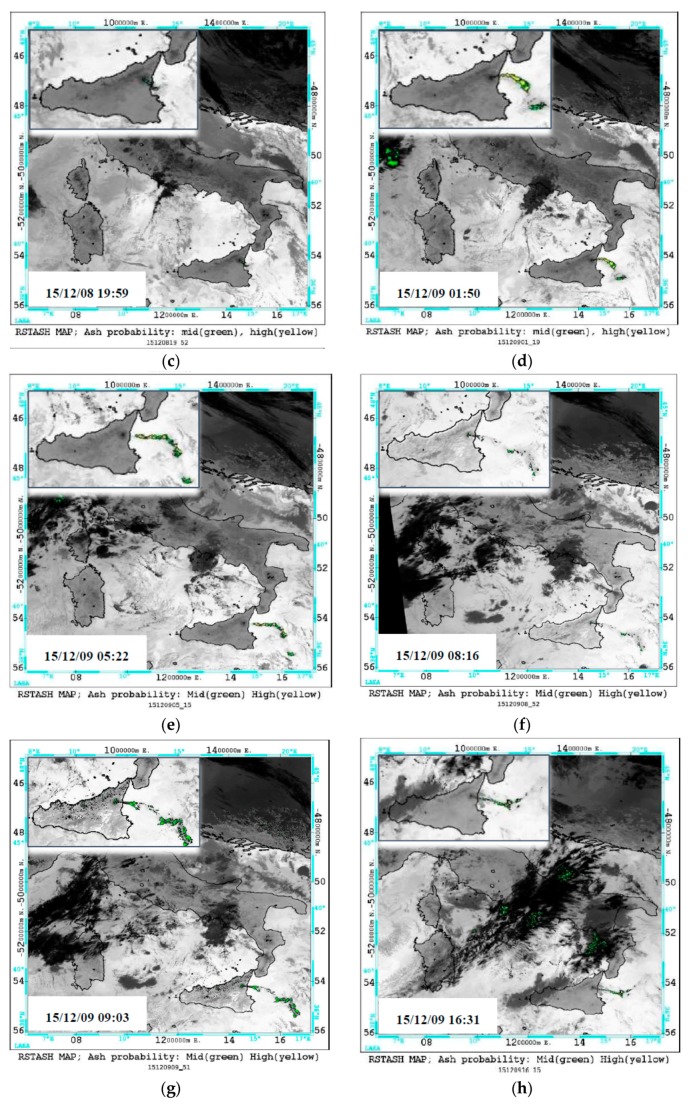

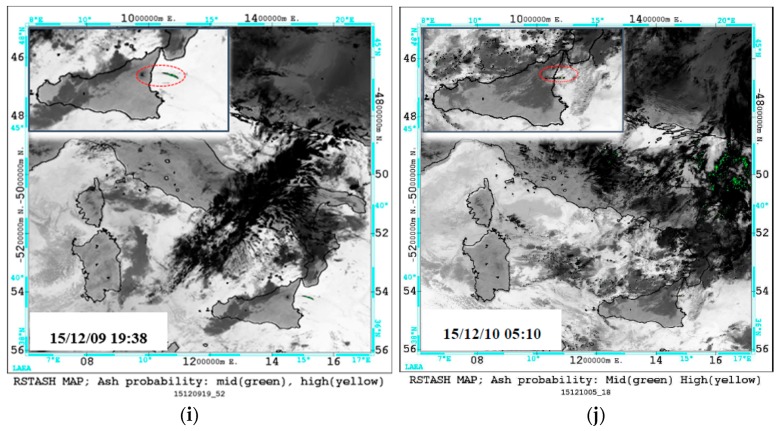
RST_ASH_ maps from AVHRR data of 8–10 December 2015 showing an ash plume (green/yellow pixels) dispersing from Mt. Etna area towards sea regions. On the top-left side of each panel, the zoom of ash-affected areas (dotted red ellipse indicates a small plume). (**a**) 8 December at 05:33 UTC; (**b**) 8 December at 08:37 UTC; (**c**) 8 December at 19:59 UTC; (**d**) 9 December at 01:50 UTC; (**e**) 9 December at 05:22 UTC; (**f**) 9 December at 08:16 UTC; (**g**) 9 December at 09:03 UTC; (**h**) 9 December at 16:31 UTC; (**i**) 9 December at 19:38 UTC; (**j**) 10 December at 05:10 UTC.

Figure 8 displays a time sequence of RST_ASH_ maps generated during 8–10 December, when the NEC emitted ash (eruption at the NSEC ended on 8 December at 05:00 UTC [4]).

Figure 8 shows that the Mt. Etna plume was initially very small (e.g., see the few ash pixels flagged within the red ellipse; the other ones represented artefacts generated in correspondence of some cloudy areas), moving from Mt. Etna towards Ionian Sea, in the East direction. Starting from the evening of 8 December, plume direction changed from E to SE; see panels (a) and (b). A few hours later, the ash plume became more evident (e.g., see panel (c)). Besides, its features probably changed in terms of ash content and/or particle size, as indicated by the increase of ash pixels associated to a high confidence level of detection (i.e., those depicted in yellow). Since the early morning of 9 December, the plume dispersed over a larger sea area (see pixels flagged at around 36° N 17° E). Afterwards, the ash BTD signal weakened (see map in panel (f) generated under high values of satellite zenith angle), and a smaller portion of the plume was identified by satellite (see panels (g) and (h) for comparison). In the early morning of 10 December, a thin and less extended ash plume affected the region of interest (ROI) (see panel (j)). In the following hours, RST_ASH_ did not provide any evidence of ash also because of limited AVHRR temporal coverage (currently 10 passages per day over Italy are available combining observations from NOAA and MetOp platforms), which did not enable the identification of intermittent/weak ash emissions that were in progress at the monitored volcano (see Section 1).

### 4.3. Estimates of Plume Height, Ash Coverage and Mass Eruption Rate

Table 1 reports values of ash coverage and plume height retrieved during 8–10 December, analyzing the same dataset of Figure 8. We estimated the plume height by comparing the minimum brightness temperature of ash pixels, measured in the 11 µm AVHRR channel, with the atmospheric temperature profiles from the NCEP (National Centers for Environmental Prediction) Reanalysis dataset. The latter provides information four times per day (i.e., at 00:00 UTC; 06:00 UTC; 12:00 UTC; 18:00 UTC) about a number of variables (e.g., air temperature, geopotential height, relative humidity) at 17 different pressures levels, with a spatial sampling of 2.5 × 2.5 degrees [47]. In more detail, we retrieved a range of ash plume top heights for each analyzed satellite scene by elevations with air temperatures closest (i.e., immediately above and below) to the minimum plume temperature measured by satellite (e.g., [48]). Table 1 shows that in the early morning of 8 December, when volcanic ash affected a very small area, the plume was at an altitude of about 3.2–4.4 km a.s.l., extending up to about 4.4–5.8 km a.s.l. during the night of the same day. Some hours later, although airborne ash affected a larger portion of the ROI, covering an area of about 1450 km^2^, plume height decreased ranging between 1.6 and 3.2 km a.s.l. This reduction in the plume altitude was possibly determined by a less efficient identification of ash pixels close to the eruptive center. Indeed, when RST_ASH_ performed better in detecting the proximal plume region we retrieved a higher value of analyzed parameter; as for data acquired since 9 December at 08:16 UTC, when plume height was in the range of 3.2–4.4 km a.s.l. 

Regarding the mass eruption rate from NEC (~3300 m a.s.l.), it was determined by using the estimated plume height (we considered only the max value retrieved on each analyzed satellite scene, because of NEC elevation, converted in km above the vent) and the empirical formulation described in Section 3.2. By results of this computation, we found that intensity of ash emissions increased during the night of 8–9 December, when the MER ≈ 6.0 × 10^3^ kg/s, fitting with information reported in a recent study [4]. 

## 5. Discussion

In this work, we have investigated the Mt. Etna plumes emitted in December 2015 by analyzing both AVHRR and SEVIRI data. 

**Figure 9:**
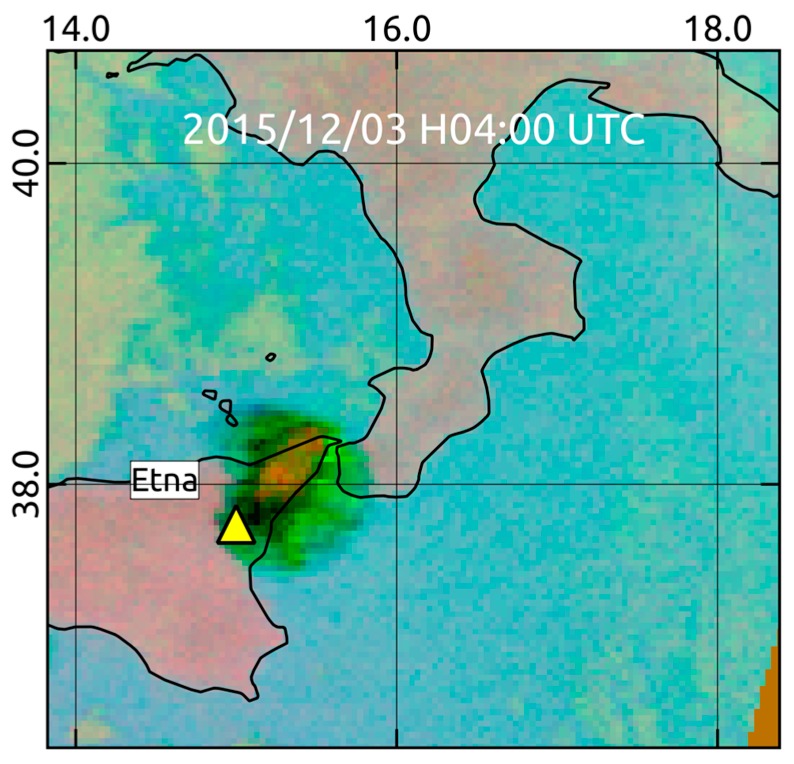
Ash RGB product from SEVIRI data of 3 December at 04:00 UTC generated according to the EUMETSAT scheme. Green pixels indicated SO_2_ areas, orange/brown pixels and dark-blue pixels respectively marked a thick and thin ice cloud.

Despite the ash emission occurring during the night of 3 December (an increase of emitted ash, travelling north-eastwards, was recorded at 02:30 UTC [49]), the operational RST_ASH_ product (which may be delivered upon request) provided information on the Mt. Etna plume about 5 h after the end of first paroxysm from VOR. Indeed, by the analysis of SEVIRI imagery temporally close to AVHRR observations of 04:20 UTC and 04:50 UTC, we found the absence of a clear ash spectral signature. On the other hand, the same analysis confirmed the presence of sulfur dioxide within the plume (e.g., [43]), indicating that RST_ASH_ probably did not detect ash due to an ice and SO_2_ cloud masking effect. This hypothesis is compatible with information provided by Figure 9, displaying the EUMETSAT ash RGB product of 3 December at 04:00 UTC. The figure shows that a thick ice cloud (see pixels in orange/brown color), bordered by volcanic SO_2_ (see green pixels), affected part of Sicily at the time of analyzed satellite observation. The SO_2_ plume emitted by Mt. Etna then separated in two different branches, as indicated also by the experimental RST-based product, tailored to SEVIRI data, for the first time used and tested here (see Figure 7b). This qualitative product, which may be generated by also exploiting infrared VIIRS (Visible Infrared Imaging Radiometer Suite) data at 750 m spatial resolution [50], seems to be sensitive to volcanic SO_2_. It should be remarked, however, that we performed only a preliminary assessment of above-mentioned RST product by means of independent GOME-2 observations. Therefore, further investigations are required for better evaluating its potential in accurately mapping SO_2_ areas under different environmental and observation conditions.

While on 3 December plume features affected RST_ASH_ detections, in the following days clouds were the major issue, preventing the identification of airborne ash by satellite (e.g., only a few AVHRR images of 6–7 December, which are not shown here, provided evidences of a weak ash emission from NSEC). On the other hand, when AVHRR data were less affected by meteorological clouds, RST_ASH_ provided more continuous information about the Mt. Etna plume (the operational usage of SEVIRI data should guarantee further improvements in this direction), successfully tracking its space-time evolution. In particular, during 8–10 December the continuity of RST_ASH_ detections allowed us to investigate temporal fluctuations in the plume height, and to retrieve the mass eruption rate from NEC; previous estimates of those parameters mainly referred to VOR paroxysms (e.g., [4,51,52]). Mild Strombolian explosions were recorded at this crater during the night of 8–9 December [4], when the plume height ranged between 4.4 and 5.8 km a.s.l., as indicated by estimates of this parameter reported in Table 1. Moreover, according to independent information, the ash plume reached on 9 December, an altitude slightly lower than 4.0 km and not higher than 5.0 km a.s.l. (Marco Neri, personal communication). Hence, estimates of plume height reported in Table 1 (a mean range of 3.2–4.4 km a.s.l. was retrieved from RST_ASH_ detections during the investigated period) appears plausible. Regarding the average MER value characterizing the eruptive activity from NEC (~1.5 × 10^3^ kg/s) derived by using Equation (3), it was compatible with a small ash eruption (e.g., [53]). In more detail, the MER retrieved here was significantly lower than that independently estimated for paroxysms of 3–5 December (e.g., [4,54]), which were among the strongest eruptive events occurring at Mt. Etna in the last 20 years [8], causing important changes of the summit area morphology [4]. 

## 6. Conclusions

Mt. Etna eruptive activity of December 2015 was particularly interesting since it involved all the summit craters. This work, integrating outcomes of previous studies, has shown the important contribution offered by the operational RST_ASH_ product in detecting ash plumes from Mt. Etna, using both nighttime and daytime AVHRR records. However, it has also confirmed main limitations (e.g., cloud coverage; possible presence of ice in volcanic clouds) affecting the near-real time monitoring of ash phenomena from space even when well-established ash detection methods are used. 

An enhanced RST_ASH_ product has recently been developed and tested in the framework of EUNADICS-AV (European Natural Airborne Disaster Information and Coordination System for Aviation) European Project to further increase efficiency of ash identification. This product should guarantee a better monitoring of ash plumes emitted by volcanoes also different from Mt. Etna, thanks to the high spatial coverage and 15 min temporal resolution of SEVIRI data, as indicated by a recent validation analysis performed by means of ground-based lidar measurements, whose results will be the aim of a next work. 

## Figures and Tables

**Table 1:** Satellite overpass times, range of plume height (i.e., min and max value for each analyzed satellite scene) and ash coverage retrieved starting from RST_ASH_ detections of 8–10 December 2015.

Satellite Overpass Time (YYMMDD_hhmm)	Plume Height Range (km a.s.l.)	Ash Coverage (km^2^)
151208_0533 151208_0837	3.2–4.4 3.2–4.4	49.4 58.8
151208_1959	4.4–5.8	172.8
151209_0150 151209_0522 151209_0816 151209_0903	4.4–5.8 1.6–3.2 3.2–4.4 3.2–4.4	1173.3 1450.7 351 1530
151209_1631	3.2–4.4	639.5
151209_1938	3.2–4.4	91.7
151210_0510	3.2–4.4	76.4
